# Impact of Hemorrhage Extent on External Ventricular Drain-Associated Infections in Aneurysmal Subarachnoid Hemorrhage

**DOI:** 10.1007/s12028-025-02310-4

**Published:** 2025-06-26

**Authors:** Florian Ebel, Emilia Westarp, Matteo Poretti, Matthias von Rotz, Simon Stohler, Raymond Chen, Raphael Guzman, Maja Weisser, Sarah Tschudin-Sutter, Luigi Mariani, Michel Roethlisberger

**Affiliations:** 1 Department of Neurosurgery, University Hospital of Basel, Basel, Switzerland; 2 Faculty of Medicine, University of Basel, Basel, Switzerland; 3 Department of Infectious Diseases, University Hospital of Basel, Basel, Switzerland; 4 Division of Pediatric Neurosurgery, University Children’s Hospital of Basel, Basel, Switzerland

**Keywords:** Aneurysmal subarachnoid hemorrhage, External ventricular drain, Nosocomial meningitis, Ventriculitis, Hemorrhage extent

## Abstract

**Background:**

External ventricular drain (EVD)–associated infections (EVDAI) remain a relevant complication of acute hydrocephalus treatment following aneurysmal subarachnoid hemorrhage (aSAH). Whether radiological quantity and anatomical distribution of subarachnoid and ventricular blood impact EVDAI rates has not been thoroughly studied to date.

**Methods:**

This was a retrospective (2009–2023) analysis of patients with aSAH undergoing emergency ventriculostomy. Univariable and multivariable logistic regression analyses were used to assess the association between the Barrow Neurological Institute (BNI) grading scale for subarachnoid hemorrhage and the intraventricular hemorrhage (IVH) score for extent and anatomical distribution of intracerebral bleeding with EVDAI risk. Cox regression analysis was employed to investigate the relationship between hemorrhage extent and the timing of EVDAI onset.

**Results:**

One hundred and ninety-four patients with aSAH received 228 EVDs with a total of cumulative 2,258 EVD days. Overall EVDAI rates were 14% (27/194) per patient and 12% (27/228) per EVD. EVDAI was associated with a larger subarachnoid blood clot (BNI grade 4; odds ratio 6.66, 95% confidence interval 2.04–21.68; *p* = 0.002) and higher IVH scores (odds ratio 1.33, 95% confidence interval 1.05–1.69; *p* = 0.02). Intracerebral hemorrhage was more frequently localized in the posterior fossa in the EVDAI group (20% vs. 0%, *p* = 0.004). Multivariable analysis confirmed a positive independent correlation with larger blood clots. Cox regression demonstrated earlier EVDAI onset in association with higher BNI grades and IVH scores.

**Conclusions:**

Both the quantity and radiological distribution of subarachnoid and ventricular blood positively correlate with EVD-associated nosocomial meningitis, eventually accelerating an earlier infection onset. These findings should help guide future research on EVDAI prevention in patients with aSAH.

**Supplementary Information:**

The online version contains supplementary material available at 10.1007/s12028-025-02310-4.

## Introduction

The insertion of an external ventricular drain (EVD) is a common neurosurgical emergency procedure primarily used to treat acute hydrocephalus and reduce intracranial pressure in aneurysmal subarachnoid hemorrhage (aSAH), and it is required in up to 50% of affected patients [1, 2]. Nosocomial meningitis and ventriculitis caused by EVD-associated infection (EVDAI) remains the most frequent EVD-related complication, occurring in approximately 3 to 9% [3–7]. This complication significantly increases patient morbidity, hospitalization time, and health care costs [3–6]. Although various studies have identified a myriad of risk factors for the development of EVDAI, the quantity and radiological distribution of subarachnoid, intracerebral, and ventricular blood have not been thoroughly analyzed as an independent predictor of EVDAI [4–6, 8–13]. Thus, this study aims to assess the extent and distribution of hemorrhage on the risk of EVDAI in patients with aSAH.

## Methods

### Patient Selection

**Fig. 1:**
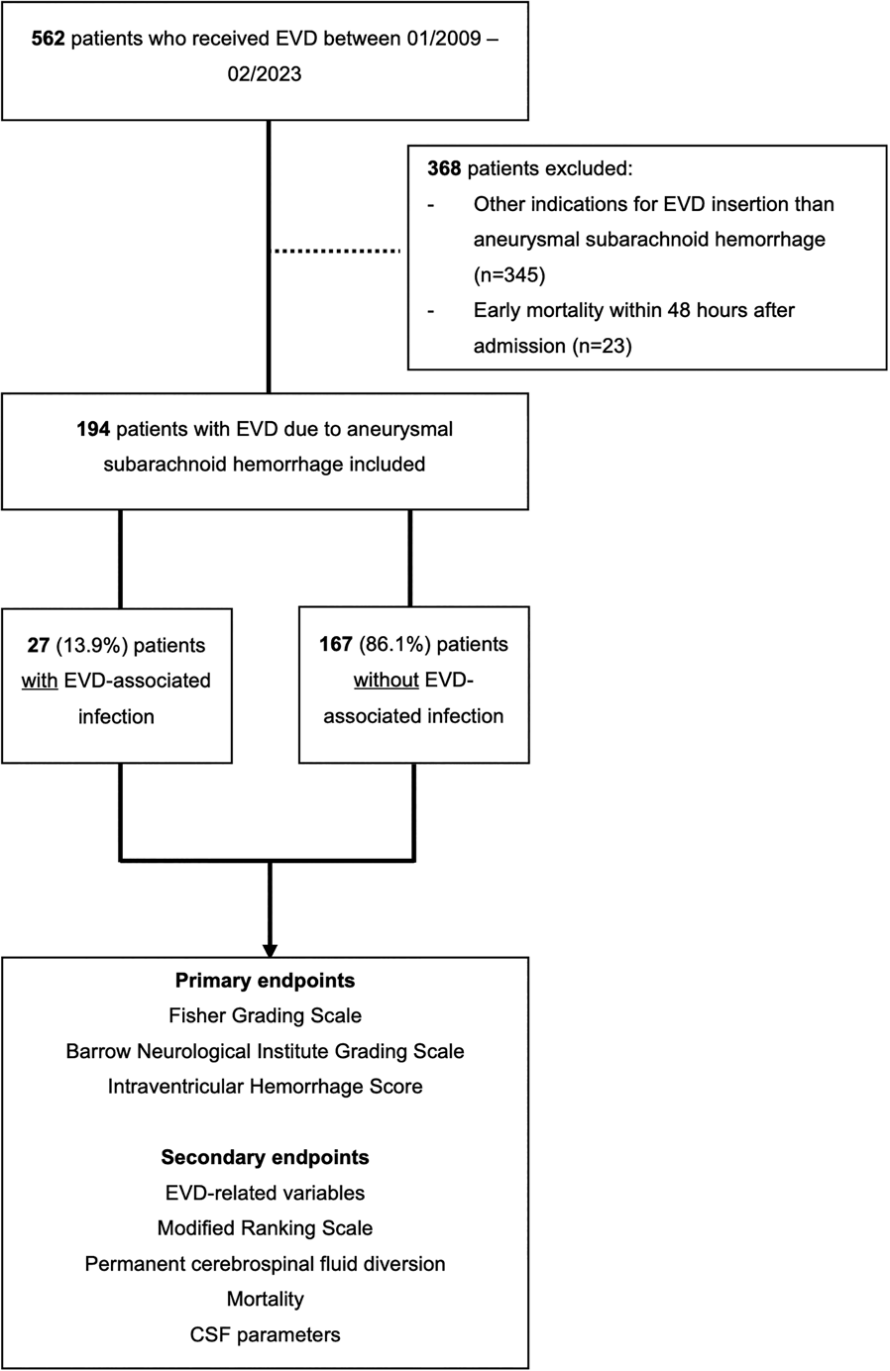
Patient selection flowchart. Of 562 patients undergoing emergency ventriculostomy between 2009–2023, 368 were excluded, leaving 194 patients with aSAH for analysis

Consecutive adult patients with aSAH with confirmed ruptured aneurysm by computed tomography angiography or digital subtraction angiography who underwent EVD placement at the Department of Neurosurgery, University Hospital Basel, between January 2009 and February 2023, were included in this retrospective single-center study. Exclusion criteria were all other indications for EVD except aSAH, patient age < 18 years, and mortality within 48 h of referral (Fig. 1). Ethical approval was obtained from the local ethics committee, and the requirement for informed consent was waived.

### Data Collection

Data were derived from both retrospectively (2009–2015 and 2019–2023) and prospectively (2016–2018) collected patient records. Predefined clinical and radiological variables were extracted from the institution’s electronic hospital information systems. Data collection was performed by MP, SS, and RC and validated by FE, EW, and MR. Hemorrhage scores (Fisher grade, Barrow Neurological Institute [BNI] Grading Scale, and intraventricular hemorrhage [IVH] score) were retrieved from radiology reports when available and reviewed for consistency; if not reported, they were independently assessed based on original imaging data.

### Definition and Outcome Measures

An EVDAI was defined based on modified criteria from the Centers for Disease Control and Prevention and the Infectious Diseases Society of America [14] Infections were classified as either “definite” if microbiological confirmation was available (positive cerebrospinal fluid [CSF] culture or gram stain) or “suspected” if diagnosis was based on clinical and biochemical findings, including (1) fever ≥ 38 °C without another identifiable source of infection and (2) CSF biochemical profile indicative of infection with declining glucose level, increasing protein level or increasing pleocytosis. All patients with EVDAI, whether definite or suspected, were independently reviewed and confirmed by infectious disease specialist and the hospital infection control team. Discrepancies were resolved through structured consensus discussion to minimize interrater variability.

The primary endpoint was the association between the extent of subarachnoid, ventricular, and intracerebral hemorrhage (ICH), based on initial imaging at presentation prior to aneurysm repair, and the occurrence of EVDAI in patients with aSAH. Subarachnoid hemorrhage was graded using the Fisher Scale (grades 1–4) and the BNI grading scale (grades 1–5) [15, 16]. The Fisher grade is defined as follows: grade 1 indicates no blood detected; grades 2 and 3 represent a layer of subarachnoid blood less than or greater than 1 mm, respectively; and grade 4 includes intraparenchymal hemorrhage or IVH with or without subarachnoid blood [16]. The BNI Grading Scale further specifies the subarachnoid blood thickness: BNI grade 1 indicates no visible subarachnoid hemorrhage, whereas BNI grades 2 to 5 represent thicknesses of ≤ 5 mm, 5–10 mm, 10–15 mm, and > 15 mm at its thickest point, respectively [15]. The extent of IVH was assessed using the IVH score by Hallevi et al. [17]. The IVH score quantifies the extent of bleeding in the right lateral ventricle and left lateral ventricle, as well as in the third ventricle and fourth ventricle separately and also describes whether hydrocephalus is present. We report the individual components of the score and the total IVH score, which ranges from 1 to 23 and is calculated using the following formula: IVH score = 3 × (right lateral ventricle + left lateral ventricle) + third ventricle + fourth ventricle + 3 × hydrocephalus [17]. For Cox regression analysis the IVH score was trichotomized (group 1: 1–7, group 2: 8–14, group 3: 15–23). ICH volume was quantified by semiautomated volumetric analysis (ml) using a segmentation tool (Sectra Workstation IDS7, Sectra, Linköping, Sweden).

Secondary endpoints were EVD-related variables such as the number, insertion side (right, left, or bilateral), replacement of the EVD, and duration of EVD. The CSF sampling quantity and whether a cutaneous chlorhexidine dressing (Tegaderm CHX I.V. Securement Dressing; 3 M GmbH, St. Paul, MN) was applied on the EVD entry site was recorded. Furthermore, EVDAI-related variables such as time point of infection onset (days) and the causative pathogen identified from sonicated catheter tips, CSF culture, and blood culture. The functional outcome at last follow-up was assessed using the modified Rankin Scale (mRS), dichotomized into a favorable (mRS score 0–2) and unfavorable (mRS score 3–5) outcome. Additional assessments included the necessity for ventriculoperitoneal shunt (VPS) insertion, early (≤ 14 days postoperatively) and late (> 14 days postoperatively) mortality rates. CSF parameters, such as leucocyte count (10e6/l), lactate (mmol/l), glucose (mmol/l) and proteins (mg/l) and the CSF/plasma ratio for lactate and glucose at baseline (first measurement), EVDAI diagnosis, were assessed at last measurement, which was conducted either shortly before EVD removal or diversion to a VPS.

### Surgical Procedure

During the study period, silver-coated EVD catheters were inserted in the operating theater and routinely used for CSF drainage rather than for intracranial pressure monitoring alone. Perioperative intravenous antibiotic prophylaxis consisted of a single dose of either cefuroxime (1.5 or 3 g) or clindamycin (600 mg). Corticosteroids were not routinely administered. Following hemicranial hair removal and a 30-s preoperative antisepsis application, EVD insertion was performed with 5 cm subcutaneous tunneling. From 2017 onward, a Tegaderm CHX I.V. Securement Dressing (3 M GmbH) was applied sterilely after skin closure, and the dressings were stapled along the edges to reduce the risk of detachment and dislocation. Postoperative care included regular CSF sampling, typically performed every 1 to 3 days, depending on the clinical context and surgeon preference. Dressing changes and antiseptic cleaning of the EVD entry site were performed every 5th to 7th day according to strict standard operating procedures [18]. Weaning was not standardized across the study period and was performed based on individual surgeon preference.

### Statistical Analysis

Statistical analysis was performed using SPSS (version 28.0, IBM Corp.). The study cohort was stratified into two groups: those who developed EVDAI (EVDAI group) and those who did not (non-EVDAI group). Continuous variables were assessed using the Mann–Whitney *U*-test or the Kruskal–Wallis test and were reported as the mean and standard deviation or the median and interquartile range (IQR). Categorical data were evaluated using the *χ*^2^ or Fisher’s exact test, depending on the variable count, and were presented as participant count with percentages. To assess predictive factors for EVDAI, a multivariable logistic regression modeling was used. Variables with statistically significant association on univariate analysis (*p* < 0.05) were included in a multivariable binary logistic regression model, and respective odds ratios (ORs) and 95% confidence intervals (CIs) are reported. To investigate the relationship between the extent of hemorrhage and time point of EVDAI onset, Cox regression analysis reporting hazard ratios (HRs) and 95% CIs was used. A *p* value < 0.05 was considered significant.

## Results

Between 2009 and 2023, 194 (35%) patients with aSAH with 228 EVDs out of 562 patients undergoing ventriculostomy were included in the analysis. Patients who received an EVD due to other indications (*n* = 345, 61%) and patients with aSAH who died within 48 h after admission (*n* = 23, 4%) were excluded (Fig. 1).

Among the 194 included patients, 27 developed an EVDAI, corresponding to an EVDAI rate per patient of 14% and per EVD of 12%. Respectively, 167 (86%) did not develop EVDAI. The EVDAI incidence per 1,000 days was 11.9. Baseline characteristics were similar between the EVDAI and non-EVDAI groups. Mean age (53.3 ± 12.2 years vs. 57.8 ± 12.7 years), sex distribution (male: 26% vs. 32%) and comorbidities were evenly distributed between the EVDAI group and the non-EVDAI group. The clinical admission scores showed no significant differences. The localization of the ruptured aneurysms and the type of treatment received (endovascular or microsurgical) were also similarly distributed. The average time from EVD insertion to EVDAI diagnosis was 9.6 ± 6.5 days. The most frequently identified pathogens were coagulase-negative staphylococci (19%) and cutibacterium acnes (19%), both detected in sonicated EVDs and CSF cultures. The CSF and blood samples were comparable between the two groups, except for the glucose level at the last CSF measurement, which was significantly lower in the EVDAI group (2.9 ± 1.1 vs. 3.7 ± 1.1, *p* = 0.001) (Supplementary Tables 1–3).

### Impact of Subarachnoid Hemorrhage Extent on EVDAI Occurrence

**Fig. 2:**
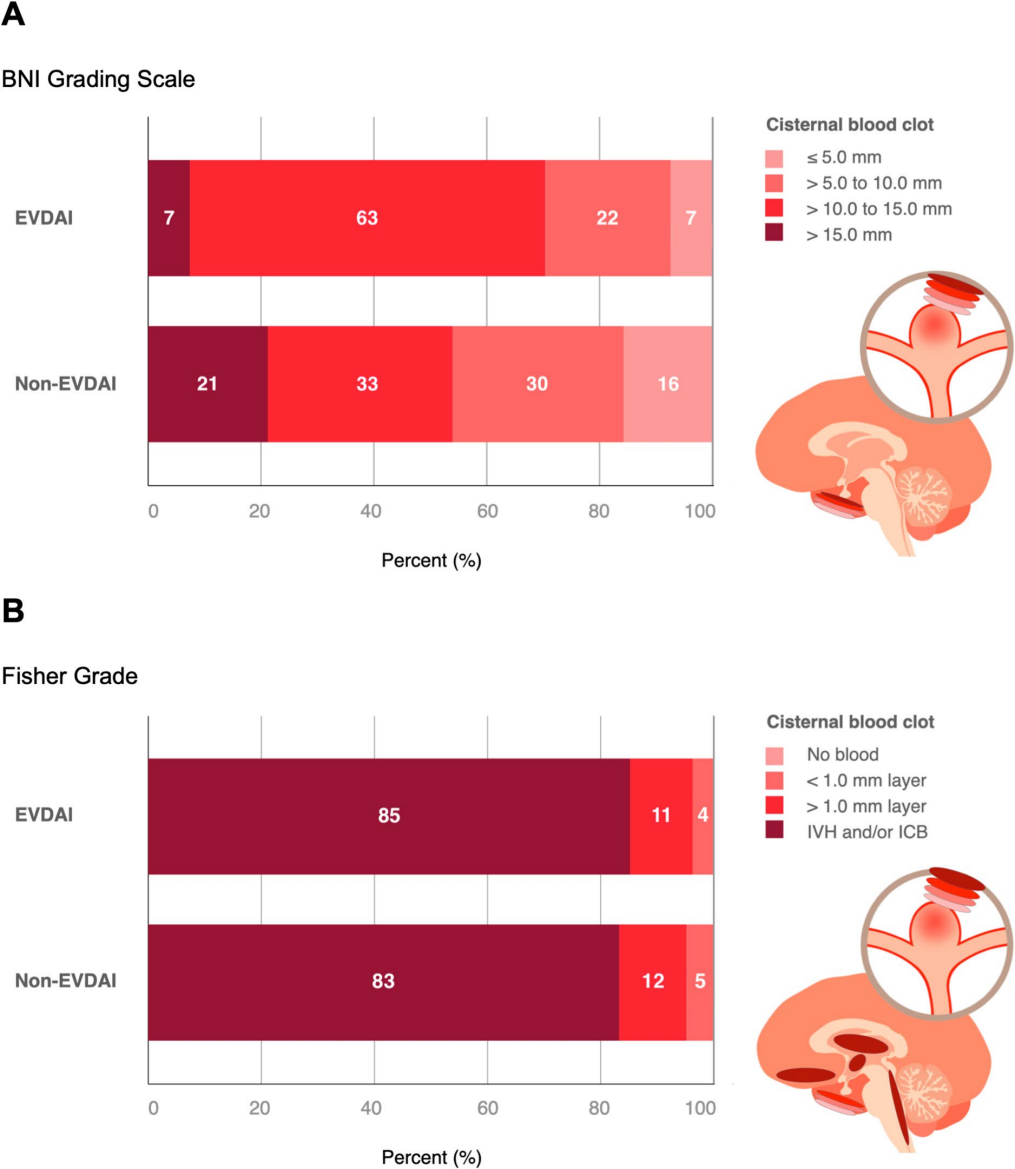
Comparison of subarachnoid hemorrhage severity and intracisternal extension on initial computed tomography in 194 aneurysmal subarachnoid patients undergoing emergency ventriculostomy, 27 of which developed EVDAI, compared to 167 patients who did not. Bar plot diagrams displaying the distribution of the respective blood clot burden (thickness) objectified by; **A** the Fisher-grading scale by Fisher et al. [16], defined as follows: Grade 1 indicates no blood detected; Grades 2 and 3 represent a layer of subarachnoid blood less than or greater than 1 mm, respectively; and Grade 4 includes intraparenchymal or intraventricular hemorrhage with or without subarachnoid blood. **B** the BNI-grading scale by Wilson et al.^15^ further specifying the cisternal blood clot thickness: BNI Grade 1 indicates no visible subarachnoid hemorrhage, while BNI Grades 2 to 5 represent thicknesses of ≤ 5 mm, 5–10 mm, 10–15 mm, and > 15 mm at its thickest point, respectively [15]. EVDAI, external ventricular drain associated infection; BNI, Barrow Neurological Institute

The extent of subarachnoid hemorrhage, measured by Fisher grade on initial radiological imaging, was comparable between the EVDAI and non-EVDAI groups. When measured by the BNI grade, which provides a more detailed evaluation of cisternal blood clot thickness, severe hemorrhages (BNI grade 4) were more frequent in the EVDAI group (63% vs. 32.7%, *p* = 0.003). Multivariable logistic regression analysis confirmed that a BNI grade 4 was a strong independent predictor for developing EVDAI (OR 6.66, 95% CI 2.04–21.68; *p* = 0.002) (Tables 1 and 2, Fig. 2).

**Table 1:** Radiological hemorrhage distribution, categorized into subarachnoid, intracerebral and intraventricular hemorrhage on initial computed tomography imaging, between aSAH patients undergoing EVD-insertion with and without nosocomial EVD-associated infection (EVDAI)

Subarachnoid Extension	Total	EVDAI Group	Non-EVDAI Group	*P* value
Fisher Grade (median, IQR)	4 (4;4)	4 (4;4)	4 (4;4)	0.792
Fisher 2	9 (4.6)	1 (3.7)	8 (4.8)	0.803
Fisher 3	23 (11.9)	3 (11.1)	20 (12)	0.897
Fisher 4	162 (83.5)	23 (85.2)	139 (83.2)	0.8
BNI Grade (median, IQR)	4 (3;4)	4 (3;4)	4 (3;4)	0.557
BNI 2	28 (14.6)	2 (7.4)	26 (15.8)	0.254
BNI 3	56 (29.2)	6 (22.2)	50 (30.3)	0.392
BNI 4	71 (37)	17 (63)	54 (32.7)	**0.003**
BNI 5	37 (19.3)	2 (7.4)	35 (21.2)	0.092
*Intracerebral Extension*				
SAH with ICH	45 (23.4)	5 (18.5)	40 (24.2)	0.515
Volume of hemorrhage (ml)	24.2 ± 19.4	6.9 ± 6.8	25.9 ± 19.5	**0.019**
Location				
Frontal	26 (57.8)	3 (60)	23 (57.5)	0.915
Temporal	14 (31.1)	–	14 (35)	0.111
Parietal	3 (6.7)	1 (20)	2 (5)	0.205
Basal ganglia	1 (2.2)	–	1 (2.5)	0.721
Posterior fossa	1 (2.2)	1 (20)	–	**0.004**
*Intraventricular Extension*				
aSAH with IVH	168 (86.6)	24 (88.9)	144 (86.2)	0.706
IVH Score (median, IQR)	10 (6;14)	11 (8;20)	10 (6;12)	0.127
Left ventricle				
No blood	67 (34.9)	9 (33.3)	58 (35.2)	0.854
< 1/3 filled with blood	82 (42.7)	9 (33.3)	73 (44.2)	0.288
1/3–2/3 filled with blood	19 (9.9)	5 (18.5)	14 (8.5)	0.106
Completely filled with blood	24 (12.5)	4 (14.8)	20 (12.1)	0.695
Right ventricle				
No blood	64 (33.3)	8 (29.6)	56 (33.9)	0.66
< 1/3 filled with blood	93 (48.4)	10 (37)	83 (50.3)	0.201
1/3–2/3 filled with blood	16 (8.3)	2 (7.4)	14 (8.5)	0.851
Completely filled with blood	19 (9.9)	7 (25.9)	12 (7.3)	**0.003**
Third ventricle with blood	132 (68.8)	21 (77.8)	111 (67.3)	0.275
Fourth ventricle with blood	137 (71.4)	23 (85.2)	114 (69.1)	0.086
Hydrocephalus	165 (85.9)	25 (92.6)	140 (84.8)	0.283

*EVDAI* external ventricular drain-associated infection, *IQR* interquartile range, *BNI* Barrow Neurological Institute, *aSAH* subarachnoid hemorrhage, *ICH* intracerebral hemorrhage, *IVH* intraventricular hemorrhage

**Table 2:** Multivariable logistic regression analysis showing the association of age, gender, EVD number, and hemorrhage extension with the development of a nosocomial EVD-associated infection (EVDAI)

Multivariable logistic regression	Odds Ratio (95% CI)	*P* value
Age	0.96 (0.92–1)	0.06
Gender (ref: male)	2.49 (0.83–7.42)	0.102
Number of EVD	2.22 (0.83–5.91)	0.111
CHX dressing (ref: no CHX dressing)	0.35 (0.13–0.92)	**0.034**
IVH-Score	1.33 (1.05–1.69)	**0.02**
Fisher Grade	0.49 (0.16–1.48)	0.208
BNI Grade	0.81 (0.39–1.7)	0.579
BNI Grade = 4 (ref: other scores)	6.66 (2.04–21.68)	**0.002**

*CI* confidence interval, *ref* reference, *EVD* external ventricular drain, *CHX* chlorhexidine, *BNI* barrow neurological institute

### Impact of ICH and IVH Extent on EVDAI Occurrence

Forty-five of 194 patients (23%) had ICH on initial radiological imaging. ICH volume was higher in the non-EVDAI group compared with the EVDAI group (25.9 ± 19.5 ml vs. 6.9 ± 6.8 ml, *p* = 0.019). ICH was more frequently localized in the posterior fossa in the EVDAI group (20% vs. 0%, *p* = 0.004).

**Fig. 3:**
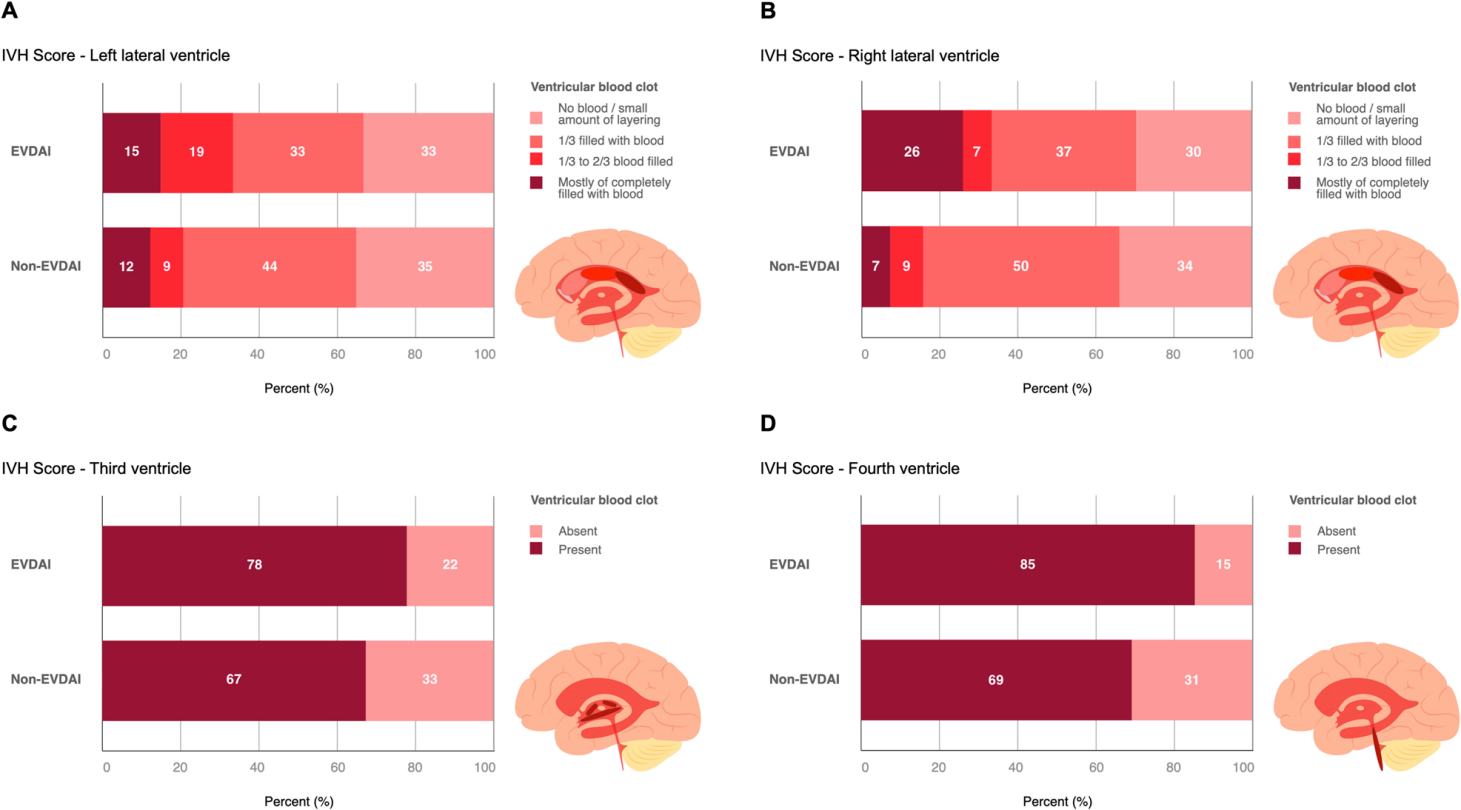
Comparison of intraventricular hemorrhage severity and ventricular extension on initial computed tomography in 194 aneurysmal subarachnoid patients undergoing emergency ventriculostomy, 27 of which developed EVDAI, compared to 167 patients who did not. Bar plot diagrams displaying the distribution of the blood clot (thickness) objectified by the IVH-score by Hallevi et al. [17]. The IVH score quantifies the extent of bleeding in the right (RV) and left lateral ventricle (LV), as well as in the third (III) and fourth ventricle (IV) separately and also describes whether hydrocephalus (H) is present. We report the individual components of the score and the total IVH score, which ranges from 1 to 23 and is calculated using the formula “IVH Score = 3 x (RV + LV) + III + IV + 3 × H”. IVH extension in the; **A** left lateral ventricle; **B** right lateral ventricle; **C** third ventricle; **D** fourth ventricle. EVDAI, external ventricular drain associated infection; IVH, intraventricular hemorrhage

In contrast, 168 of 194 (87%) patients with aSAH had an IVH. Complete filling of the right lateral ventricle was more common in the EVDAI group (26% [*n* = 7]) compared with the non-EVDAI group (7% [*n* = 12]; *p* = 0.003). Multivariable analysis confirmed that a higher IVH score was a significant risk factor for EVDAI (OR 1.33, 95% CI 1.05–1.69; *p* = 0.02). In a subgroup analysis of patients with complete filling of the right lateral ventricle (*n* = 19), 14 patients (74%) had an EVD placed into the blood-filled right ventricle, and 6 of these 14 patients (43%) developed EVDAI. Among the five patients with EVDs placed contralaterally, only one patient (20%) developed EVDAI (Tables 1 and 2, Fig. 3).

### Impact of Hemorrhage Extent on the Timing of EVDAI Onset

**Fig. 4:**
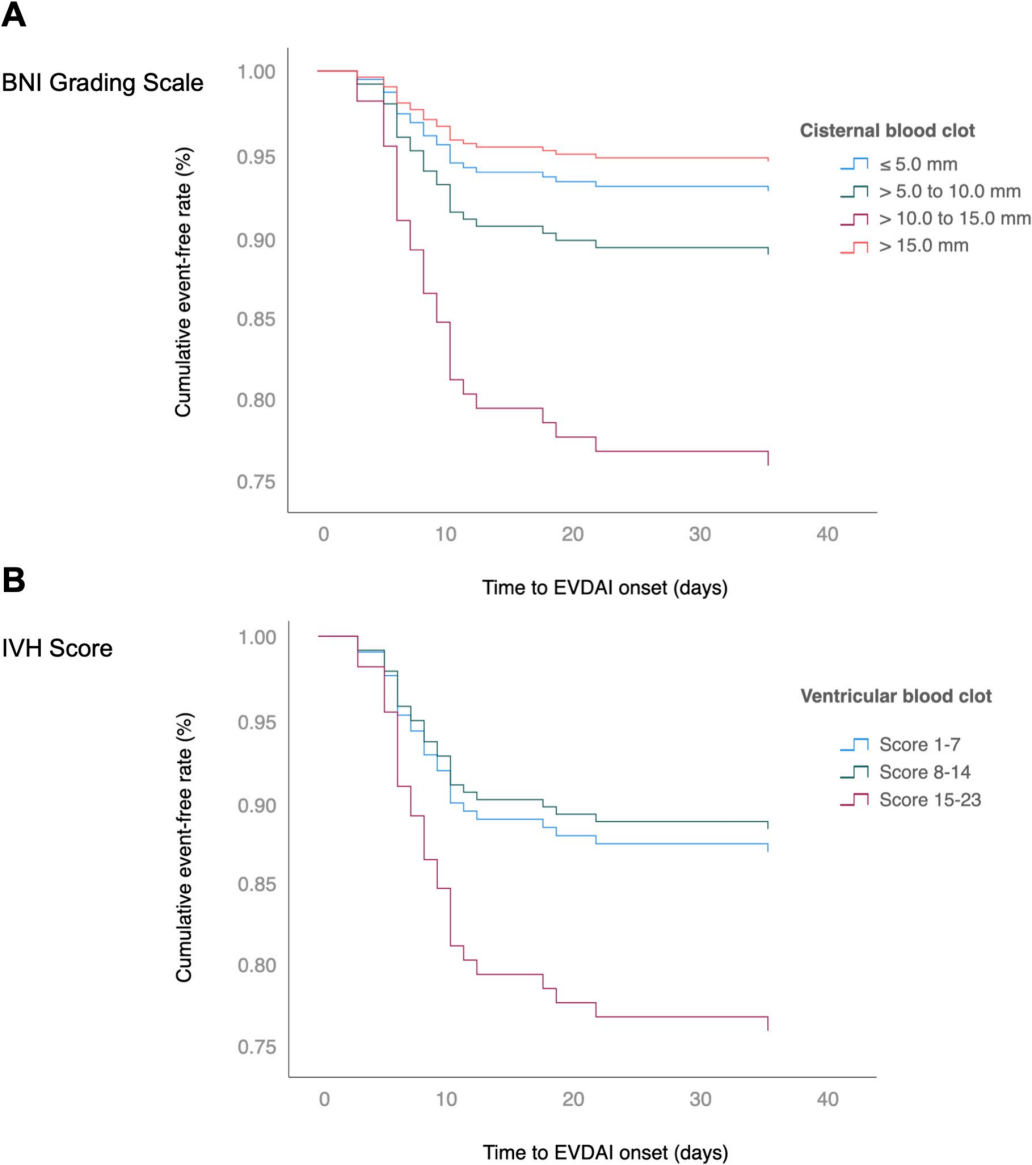
Kaplan–Meier analysis displaying the time from emergency ventriculostomy to EVDAI onset (in days) stratified for the; **A** BNI-grading scale by Wilson et al. [15]; **B** IVH-score by Hallevi et al. [17]. EVDAI, external ventricular drain associated infection; BNI, Barrow Neurological Institute; IVH, intraventricular hemorrhage

A higher BNI grade was significantly associated with an earlier onset of EVDAI. In particular, a BNI grade of 4 (HR 4.83, 95% CI 1.12–20.95; *p* = 0.035) was found to be associated with an earlier occurrence. In accordance, an increased IVH score was shown to be associated with an earlier occurrence of EVDAI (HR 1.2, 95% CI 1.01–1.43; *p* = 0.049) (Fig. 4).

### Impact of EVD-Related Variables on EVDAI Occurrence

A total of 228 EVDs were inserted, with a higher EVD-per-patient rate in the EVDAI compared with the non-EVDAI group (1.4 ± 0.6 vs. 1.1 ± 0.4, *p* = 0.01). Multiple EVD insertions were more frequent in the EVDAI group compared with the non-EVDAI group (33% vs. 14%, *p* = 0.01), whereas EVD replacement rates tended to be higher without reaching statistical significance (18.5% vs. 8.4%, *p* = 0.1). Initial EVD insertion was predominantly on the right side in both groups (67% vs. 74%). Cutaneous dressings containing chlorhexidine-gluconate protecting the EVD entry site were more commonly used in the non-EVDAI group (62% vs. 37%, *p* = 0.016) and were associated with a significantly reduced risk of EVDAI (OR 0.35, 95%CI [0.13–0.92]; *p* = 0.034). The EVD in-situ duration was comparable between groups, with an average CSF sampling rate of 11 ± 5.6 in the EVDAI group compared with 9.3 ± 5.1 in the non-EVDAI group (Tables 2 and 3).

**Table 3:** Details of EVD Management between aSAH patients undergoing EVD-insertion with and without nosocomial EVD-associated infection (EVDAI)

EVD details	Total	EVDAI Group	Non-EVDAI Group	*P* value
Total No. of EVD	228	37	191	
No. of EVD per patient (mean ± SD)	1.2 ± 0.4	1.4 ± 0.6	1.1 ± 0.4	**0.01**
Patients with single EVD	162	18 (66.7)	144 (86.2)	**0.01**
Patients with multiple EVDs*	32	9 (33.3)	23 (13.8)	**0.01**
Side of 1st EVD				0.314
Right	141 (72.7)	18 (66.7)	123 (73.7)	
Left	43 (22.2)	6 (22.2)	37 (22.2)	
Bilateral	10 (5.2)	3 (11.1)	7 (4.2)	
Replacement of initial EVD	19	5 (18.5)	14 (8.4)	0.1
Chlorhexidine dressing	107	10 (37)	97 (61.8)	**0.016**
Cummulative EVD duration (days)	2258	317	1941	
EVD duration per patient (mean ± SD)†	11.6 ± 6.5	11.7 ± 7.5	11.6 ± 6.3	0.623
CSF sampling (mean ± SD)	9.5 ± 5.2	11 ± 5.6	9.3 ± 5.1	0.256

*aSAH* subarachnoid hemorrhage, *EVDAI* external ventricular drain-associated infection, *EVD* external ventricular drain, *No*. number, *SD* standard deviation

*Multiple EVDs include initial bilateral EVD placements, sequential contralateral insertions, or replacement of the initial EVD due to misplacement, suspected infection, or obstruction

†In patients with multiple EVDs, the durations were summed to calculate the EVD duration per patient

### Clinical Outcome and Ventriculoperitoneal Shunt-Rates

The functional outcome at last follow-up (57.4 ± 38.4 months) measured by the median (IQR) mRS was comparable between the groups. The overall mortality rate was 31% (*n* = 59), with comparable mortality rates between the EVDAI and non-EVDAI groups. Early mortality (≤ 14 days) appeared in 2 (29%) and 29 (56%) patients in the EVDAI and non-EVDAI groups, respectively. Seventy-eight of the 194 patients needed a VPS insertion, resulting in an overall VPS rate of 40%. VPS rates were slightly higher in the EVDAI group (12/27 [44%]) compared with the non-EVDAI group (66/164 [40%]), respectively (Table 4).

**Table 4:** Clinical outcomes, CSF-diversion rates, and mortality rates between aSAH patients undergoing EVD-insertion with and without nosocomial EVD-associated infection (EVDAI)

	Total	EVDAI Group	Non-EVDAI Group	*P* value
Length of stay (mean ± SD)	21.8 ± 9.5	23.7 ± 6.5	21.5 ± 9.9	0.149
*Clinical condition at last Follow-up*				
mRS (median, IQR)	2 (1;3)	2 (1;3)	2 (1;3)	0.275
mRS grouped				0.22
Favourable (mRS 0–2)	65 (69.1)	9 (56.3)	56 (71.8)	
Unfavourable (mRS 3–5)	29 (30.9)	7 (43.8)	22 (28.2)	
Time to last FU (mths, mean ± SD)	57.4 ± 38.4	70.9 ± 42.8	54.5 ± 37	0.125
*Mortality*				
Mortality	59 (30.4)	7 (25.9)	52 (31.1)	0.735
Time to mortality				0.176
Early mortality (≤ 14 days)	31 (52.5)	2 (28.6)	29 (55.8)	
Late mortality (> 14 days)	28 (47.5)	5 (71.4)	23 (44.2)	
*Ventriculoperitoneal Shunt*				
VPS Insertion	78 (40.2)	12 (44.4)	66 (39.5)	0.628
Time to VPS insertion (days, mean ± SD)	19 ± 12.9	21.7 ± 13.7	18.5 ± 12.7	

*CSF* Cerebrospinal fluid, *EVDAI* external ventricular drain-associated infection, *SD* standard deviation, *mRS* modified Rankin Scale, *IQR* interquartile range, *FU* follow-up, *mths* months, *VPS* ventriculoperitoneal shunt

## Discussion

The present study, which assessed 194 patients with aSAH who underwent emergency EVD insertion, highlights the critical impact of hemorrhage extent in the development of EVDAI. Both the quantity and radiological distribution of subarachnoid and ventricular blood, measured by the BNI and IVH score, were associated with an increased risk and earlier onset of EVDAI. Our findings have important clinical implications for managing patients with aSAH requiring EVD placement.

Aneurysm rupture typically results in subarachnoid blood distribution within the basal, interhemispheric and sylvian cisterns. IVH and ICH are observed in up to 50% and 21% of patients with aSAH, respectively [19–21]. Approximately 50% of patients with aSAH require EVD placement, often related to the extent of subarachnoid hemorrhage and IVH [11, 22]. In our cohort, these figures were even higher, with 87% of patients with aSAH presenting with IVH, and 23% with ICH. This highlights the pronounced hemorrhage burden in our patients with aSAH requiring EVD placement.

In this context, the EVDAI rate in our study was 14% per patient and 12% per EVD. Although this exceeds the rates of 3–9% typically reported in unselected neurosurgical populations, it may reflect the higher baseline risk of our aSAH cohort [3–7]. All infections were defined using strict criteria and independently confirmed by infectious disease specialists, suggesting a high level of diagnostic accuracy. As such, our findings may provide a more accurate estimate for EVDAI risk in the context of aSAH. Although various risk factors for EVDAI have been described, no prior study has assessed the extent of hemorrhage following aneurysm rupture as an independent predictor for EVDAI [4–6].

Our study showed that the subarachnoid hemorrhage extent, objectified by the BNI grade, was a predictor for EVDAI. A BNI grade of 4 was associated with a sixfold increased risk (OR 6.66, 95% CI 2.04–21.68; *p* = 0.002), whereas a BNI grade 5 showed no association. The lack of association may be explained by the high early mortality rate in this group, which likely limited observation period for EVDAI to manifest. The mean time to infection onset in our cohort was 9.6 days, consistent with prior reports indicating peak incidence between days 5 and 14 (Supplementary Table 3) [2, 8, 13, 23, 24]. This finding emphasizes the complex interplay between hemorrhage severity, early mortality, and EVDAI risk, suggesting that although extensive hemorrhages increase susceptibility to infection, they may also shorten the temporal window in which EVDAI can clinically develop. This is supported by the pathophysiology of nosocomial meningitis, which is more likely to occur via retrograde catheter colonization over time, in contrast to community-acquired meningitis [18, 25, 26]. In line with this, higher BNI grades were also associated with earlier onset of EVDAI in our cohort (Fig. 4).

Similarly, the IVH extent, measured by the IVH score, emerged as a significant predictor for EVDAI (OR 1.33, 95% CI 1.05–1.69; *p* = 0.02). Patients with higher IVH scores showed a significantly increased risk of EVDAI and experienced earlier onset of infection. Interestingly, complete filling of the right lateral ventricle was more common in the EVDAI group (26% vs. 7%, *p* = 0.003). In a subgroup analysis, patients with EVD placement into a blood-filled right ventricle had a higher rate of EVDAI (43%) compared with those with contralateral placement (20%). This suggests that inserting an EVD into a blood-filled ventricle may increase infection risk. Whether contralateral placement could mitigate this remains an open question and warrants further research.

In sum, our findings suggest that the extent and distribution of subarachnoid hemorrhage and IVH correlate with both the risk and the timing of EVDAI onset. Several pathophysiological mechanisms may explain this association: increased levels of proteinaceous and hemolytic degradation products, impaired CSF dynamics, and prolonged need for CSF drainage, all of which can promote inflammation, immunosuppression, and retrograde catheter colonization [4, 6, 18, 25, 27–30]. Larger blood clots within the subarachnoid and ventricular spaces offer favorable environment for microbial growth and may increase the risk of EVD obstruction and manipulation, further contributing to infection risk [5, 31]. In contrast, the presence of ICH was not associated with increased EVDAI risk or earlier onset in our study, reinforcing the relevance of CSF-compartment burden specifically.

These observations underline the need for targeted infection prevention strategies in high-risk patients with aSAH with extensive subarachnoid and IVH. Despite the widespread use of EVDs in neurosurgical practice, significant variation exists in perioperative and postoperative management. The decision on where to place the EVD, particularly in the presence of intraventricular blood, may need to be revisited to optimize outcomes and reduce infection rates. Well-studied predictive factors for EVDAI include the duration of drainage, the number of EVDs inserted, and the CSF sampling rate [4–6, 18, 28, 32]. Consistent with previous literature, our study revealed a higher number of inserted EVDs per patient and a higher rate of multiple EVDs in the EVDAI group (33%) compared with the non-EVDAI group (14%). Although EVD replacement rates also tended to be higher in the EVDAI group (18.5% vs. 8.4%), this difference did not reach statistical significance. However, neither EVD duration nor the amount of CSF sampling was significantly higher in the EVDAI group, contrasting with prior studies [2, 18, 28, 32]. This may reflect limited variability in these parameters within our aSAH population, in which prolonged drainage is often maintained prophylactically due to vasospasm risk. This uniformity, combined with the relatively small number of EVDAI events (*n* = 27), may have reduced the power to detect significant associations.

Importantly, multivariate analysis revealed the use of chlorhexidine-gluconate–containing dressings at the EVD entry site as a strong protective factor for EVDAI, in line with previous studies and further corroborating our elucidated pathomechanism on how a larger extent of hemorrhages contributes to an increased risk and to an earlier onset of nosocomial EVD-associated meningitis [18, 33]. Our findings have already increased clinical awareness at our institution regarding EVDAI risk in patients with extensive hemorrhage burden. Although no formal changes to EVD protocols have yet been implemented, these results underscore the need for prospective studies to confirm our findings and guide future EVD management strategies.

### Limitations

This retrospective study is subject to all the limitations of data collection inherent to this kind of study design. Second, the findings may not be generalizable to other institutions with different patient populations, surgical techniques, or EVD management protocols. Third, although we accounted for potential confounders in our multivariable analyses, unmeasured factors, such as variations in surgical technique, EVD management practices or patient comorbidities, may still have influenced the results. Fourth, although our study focused on hemorrhage burden at presentation, the temporal evolution of subarachnoid and intraventricular blood may influence infection risk. As we did not collect longitudinal radiological data, we were unable to explore these dynamics. In addition, we did not quantify the amount of blood removed during neurosurgical clipping procedures. Intraoperative removal of intraparenchymal, subarachnoid, or intraventricular blood may reduce clot burden and thus potentially influence infection risk. Moreover, data on other infections requiring antimicrobial treatment were not systematically collected, which may have limited our ability to account for potential confounding effects.

## Conclusions

This present study demonstrates a strong positive correlation of both the quantity and radiological distribution of subarachnoid and ventricular blood with EVD-associated nosocomial meningitis and ventriculitis, eventually accelerating an earlier infection onset in patients with aSAH. Our results underscore the need for heightened infection prevention measures and may add valuable information for future guidelines on EVD placement strategies and recommendations to mitigate infection risks. Further prospective studies are needed to confirm these associations and improve management strategies for this high-risk patient population.

## Supplementary Information

Below is the link to the electronic supplementary material.

Supplementary file1 (DOCX 27 KB)
