# Supplementary material for: Impact of Hemorrhage Extent on External Ventricular Drain-Associated Infections in Aneurysmal Subarachnoid Hemorrhage
Source: Neurocrit Care. 2025 Jun 26;44(1):191–202. doi: 10.1007/s12028-025-02310-4 (PMC12819451; doi:10.1007/s12028-025-02310-4)
Supplement: Supplementary file 1 — Supplementary file1 (DOCX 27 KB) [file 12028_2025_2310_MOESM1_ESM.docx]

**Supplementary Table 1**: Baseline characteristics of both groups

|  | **Total** | **EVDAI Group** | **Non-EVDAI Group** | **P value** |
| --- | --- | --- | --- | --- |
| No. of Patients | 194 | 27 (13.9) | 167 (86.1) |  |
| Male (%) | 61 (31.4) | 7 (25.9) | 54 (32.3) | 0.506 |
| Age (mean ± SD) | 57.2 ± 12.7 | 53.3 ± 12.2 | 57.8 ± 12.7 | 0.089 |
| **Comorbidities** |  |  |  |  |
| BMI (mean ± SD) | 26.1 ± 5.7 | 27.4 ± 6.3 | 25.9 ± 5.6 | 0.216 |
| Hypertension | 82 (42.3) | 12 (44.4) | 70 (41.9) | 0.805 |
| Nicotine | 67 (34.5) | 12 (44.4) | 55 (32.9) | 0.243 |
| Diabetes mellitus | 11 (5.7) | - | 11 (6.6) | 0.17 |
| Dyslipidemia | 11 (5.7) | 3 (11.1) | 8 (4.8) | 0.188 |
| Immunodeficiency | 7 (3.6) | - | 7 (4.2) | 0.279 |
| Brain tumor | 2 (1) | - | 2 (1.2) | 0.568 |
| **Clinical condition at admission** |  |  |  |  |
| mRS (median, IQR) | 5 (3;5) | 5 (3;5) | 5 (3;5) | 1 |
| mRS grouped |  |  |  | 0.355 |
| Favourable (mRS 0-2) | 8 (4.1) | 2 (7.4) | 6 (3.6) |  |
| Unfavourable (mRS 3-5) | 186 (95.9) | 25 (92.6) | 161 (96.4) |  |
| Hunt and Hess scale (median, IQR) | 3 (2;5) | 4 (2;5) | 3 (2;5) | 0.988 |
| WFNS scale (median, IQR) | 4 (2;5) | 5 (2;5) | 3.5 (2;5) | 0.227 |
| **Aneurysm location** |  |  |  | 0.67 |
| Anterior communicating artery | 74 (38.1) | 13 (48.1) | 61 (36.5) |  |
| Middle cerebral artery | 33 (17) | 1 (3.7) | 32 (19.2) |  |
| Posterior communicating artery | 22 (11.3) | 3 (11.1) | 19 (11.4) |  |
| Internal carotid artery | 18 (9.3) | 2 (7.4) | 16 (9.6) |  |
| Basilar artery | 12 (6.2) | 3 (11.1) | 9 (5.4) |  |
| Posterior inferior cerebellar artery | 14 (7.2) | 2 (7.4) | 12 (7.2) |  |
| Anterior cerebral artery | 8 (4.1) | 1 (3.7) | 7 (4.2) |  |
| Vertebral artery | 5 (2.6) | - | 5 (3) |  |
| Posterior cerebral artery | 3 (1.5) | 1 (3.7) | 2 (1.2) |  |
| Multiple aneurysms | 3 (1.5) | 1 (3.7) | 2 (1.2) |  |
| Superior cerebellar artery | 1 (0.5) | - | 1 (0.6) |  |
| **Aneurysm treatment** |  |  |  | 0.226 |
| Endovascular management | 124 (63.9) | 21 (77.8) | 103 (61.7) |  |
| Microsurgical management | 55 (28.4) | 4 (14.8) | 51 (30.5) |  |
| No treatment | 15 (7.7) | 2 (7.4) | 13 (7.8) |  |
| Surgery duration (min; mean ± SD)* | 72.9 ± 86.1 | 59.2 ± 45.2 | 75.2 ± 91 | 0.194 |

*EVDAI = external ventricular drain-associated infection, SD = standard deviation, BMI = body mass index, mRS = modified Rankin Scale, IQR = interquartile range, WFNS = world federation of neurological surgeons.*

**Refers only to open surgical procedures and includes EVD insertion, with or without aneurysm clipping. In some cases, EVD placement and clipping were performed in separate surgeries. The duration of endovascular treatment is not considered.*

**Supplementary Table 2**: CSF and blood values at baseline, at EVDAI diagnosis, and before EVD removal between the two groups

|  | **Total** | **EVDAI Group** | **Non-EVDAI Group** | **P value** |
| --- | --- | --- | --- | --- |
| Time to first CSF sampling (d, mean ± SD) | 2.1 ± 3.6 | 2.5 ± 6 | 2.1 ± 3.1 | 0.468 |
| **Baseline values** |  |  |  |  |
| CSF |  |  |  |  |
| WBC | 437 ± 1207 | 318 ± 356 | 456 ± 1292 | 0.659 |
| Lactate | 3.2 ± 1.5 | 3.4 ± 1.5 | 3.2 ± 1.5 | 0.363 |
| Glucose | 4 ± 1 | 3.9 ± 0.7 | 4.1 ± 1.1 | 0.37 |
| Protein | 2348 ± 3100 | 2147 ± 1759 | 2380 ± 3267 | 0.35 |
| Blood |  |  |  |  |
| Glucose | 14.1 ± 69.9 | 6.7 ± 1.2 | 15.2 ± 74.9 | 0.426 |
| CSF/Blood glucose ratio | 0.6 ± 0.3 | 0.6 ± 0.1 | 0.6 ± 0.3 | 0.957 |
| **At EVDAI diagnosis** |  |  |  |  |
| CSF |  |  |  |  |
| WBC | - | 3548 ± 16342 | - | NA |
| Lactate | - | 3.7 ± 1.9 | - | NA |
| Glucose | - | 3.6 ± 1.5 | - | NA |
| Protein | - | 909 ± 752 | - | NA |
| Blood |  |  |  |  |
| Glucose | - | 7.7 ± 3.7 | - | NA |
| CSF/Blood glucose ratio | - | 0.5 ± 0.2 | - | NA |
| **Last CSF sampling** |  |  |  |  |
| Time to last CSF sampling (d, mean ± SD) | 9.9 ± 5.5 | 11.5 ± 6.9 | 9.6 ± 5.2 | 0.23 |
| CSF |  |  |  |  |
| WBC | 689 ± 5740 | 3055 ± 14708 | 272 ± 798 | 0.498 |
| Lactate | 3.2 ± 1.1 | 3.8 ± 1.8 | 3.1 ± 1.6 | 0.096 |
| Glucose | 3.6 ± 1.1 | 2.9 ± 1.1 | 3.7 ± 1.1 | **0.001** |
| Protein | 718 ± 781 | 1044 ± 1477 | 661 ± 569 | 0.13 |
| Blood |  |  |  |  |
| Glucose | 6.9 ± 2.2 | 6 ± 2.1 | 7.1 ± 2.2 | 0.299 |
| CSF/Blood glucose ratio | 0.7 ± 0.6 | 0.5 ± 0.5 | 0.7 ± 0.6 | 0.115 |

CSF = cerebrospinal fluid, d = day, SD = standard deviation, WBC = white blood cells, EVDAI = = external ventricular drain-associated infection.

**Supplementary Table 3:** Rate of EVD-associated infections per patient and per EVD, with details on microbiological analyses.

| **Variables** | **Value** |
| --- | --- |
| **EVD-associated infections** |  |
| No. of Patients with EVDAI | 27 |
| EVDAI rate per patient | 13.9% |
| EVDAI rate per EVD | 11.8% |
| Time to EVDAI (days, mean ± SD) | 9.6 ± 6.5 |
| **Microbiology** |  |
| Sonication EVD |  |
| CNS | 5 (18.5) |
| Cutibacterium acnes | 5 (18.5) |
| Staphylococcus aureus | 1 (3.7) |
| Other grampostitive rods | 1 (3.7) |
| CSF culture |  |
| CNS | 13 (48.1) |
| Streptococcus species | 4 (14.8) |
| Cutibacterium acnes | 4 (14.8) |
| Staphylococcus aureus | 3 (11.1) |
| Gramnegative rods | 3 (11.1) |
| Fungi | 2 (7.4) |
| Other grampositive rods | 1 (3.7) |
| Blood culture |  |
| CNS | 7 (25.9) |
| Gramnegative rods | 2 (7.4) |
| Fungi | 2 (7.4) |
| Staphylococcus aureus | 2 (7.4) |
| Other | 1 (3.7) |

EVD = external ventricual drain, EVDAI = = external ventricular drain-associated infection, CNS = coagulase-negative staphylocci,, SD = standard deviation, CSF = cerebrospinal fluid
